# Comparing the long-term efficacy of standard and combined minimally invasive procedures for unresectable HCC: a mixed treatment comparison

**DOI:** 10.18632/oncotarget.13145

**Published:** 2016-11-07

**Authors:** Jianghai Zhao, Hui Zhang, Lunshou Wei, Shuping Xie, Zhimin Suo

**Affiliations:** ^1^ Department of Gastroenterology, Huaihe Hospital of Henan University, Kaifeng, Henan, 475000, China

**Keywords:** unresectable hepatocellular carcinoma, transarterial chemoembolization, long-term efficacy, network meta-analysis

## Abstract

A small proportion of hepatocellular carcinoma (HCC) patients are suitable for surgical resections and various minimally invasive procedures have been introduced as alternatives to surgical resections. However, the relative efficacy of minimally invasive procedures remains to be studied in the current literature. Several popular minimally invasive procedures (monotherapy or combined therapies) were selected for comparison and their relative long-term efficacy were determined by using the statistics of hazard ratio (HR) which evaluates the survival status of HCC patients in one, two, three and four years, respectively. Evidence were obtained from the current literature and synthesized by using the approach of conventional pairwise meta-analysis and network meta-analysis (NMA). Moreover, selected minimally invasive procedures were ranked according to their surface under the cumulative ranking curve (SUCRA) which was produced by NMA in conjunction with the Markov Chain Monte Carlo (MCMC) sampling method. HCC patients treated by combined minimally invasive procedures, particularly transcatheter arterial chemoembolization (TACE) + high intensity focused ultrasound (HIFU), TACE + radiofrequency ablation (RFA), TACE + radiotherapy (RT) and TACE + Sorafenib (SOR) exhibited a significant decrease in the HR compared to those with standard TACE (HR < 1). The combined minimally invasive procedure of TACE + HIFU appears to be the most preferable therapy. PEI seems to be less favorable than other minimally invasive procedures. Combined minimally invasive procedures may be more preferable than standard minimally invasive procedures. Percutaneous ethanol injection (PEI) may not provide adequate efficacy compared to other minimally invasive procedures for unresectable HCC patients.

## INTRODUCTION

Hepatocellular carcinoma (HCC) is one of the five most common cancers worldwide and it is a prototype of inflammation-associated malignancies [1]. Although surgical operations have been prioritized for HCC patients, complete resection is not appropriate for patients who are diagnosed in advanced stages [2]. On the other hand, transarterial chemoembolization (TACE) is a standard minimally invasive procedure developed for HCC patients who are not eligible for complete resection [3]. TACE involves the injection of a chemotherapeutic agent, which induces selective vascular embolization and blocks the arteries, hence triggering tumor infarction and necrosis [4, 5]. The partial response rate for unresectable HCC patients with TACE is approximately 62% and TACE is able to prominently suppress tumor vascular invasion and progression [4]. However, TACE has its own limitations [6], for instance, TACE may further affect liver functions and damage the hepatic arterial system. As a result of this, TACE is not appropriate for patients with poor liver functions, particularly those with cirrhosis [3, 7].

Recently, new approaches have been introduced as either standard minimally invasive procedures or adjuvant therapies in order to improve the survival status of HCC patients: yttrium-90 radioembolization (TARE-90Y), radiotherapy (RT), percutaneous acetic acid injection (PAI), external-beam radiation therapy (EBRT), drug-eluting beads-transcatheter arterial chemoembolization (DEB-TACE), percutaneous ethanol injection (PEI) and sorafenib (SOR) [8–13]. Studies in the current literature suggest that standard minimally invasive procedures in combination with adjuvant therapies may be more efficacious than monotherapy. For instance, Li *et al*. concluded in their study that patients with TACE + high-intensity focused ultrasound (HIFU) exhibited significantly higher survival rate compared to those with TACE [14]. Moreover, evidence also indicated that combined strategy of TACE + PEI/RFA is more efficacious than the monotherapy of TACE with respect to long term survival rates [15]. Besides that, the combination therapy of TACE with radiofrequency ablation (RFA) may have several theoretical advantages compared to RFA alone, and patients with TACE-RFA exhibited a higher overall survival rate compared to those with monotherapy [6]. Furthermore, combining TACE with RT may trigger synergistic effects and enhance the efficacy of monotherapy [16]. However, the lack of a systematic review inspired us to compare standard minimally invasive procedures with combined therapies in order to benefit patients with unresectable HCC.

This study extended the scope of conventional meta-analysis by incorporating indirect evidence that can be obtained from clinical trials. It is anticipated that using this approach enabled us to determine the relative efficacy of standard or combined minimally invasive procedures without concerning about the ethical issues resulted from designing new randomized clinical trials.

## RESULTS

### Baseline characteristics of the included studies

**Figure 1:**
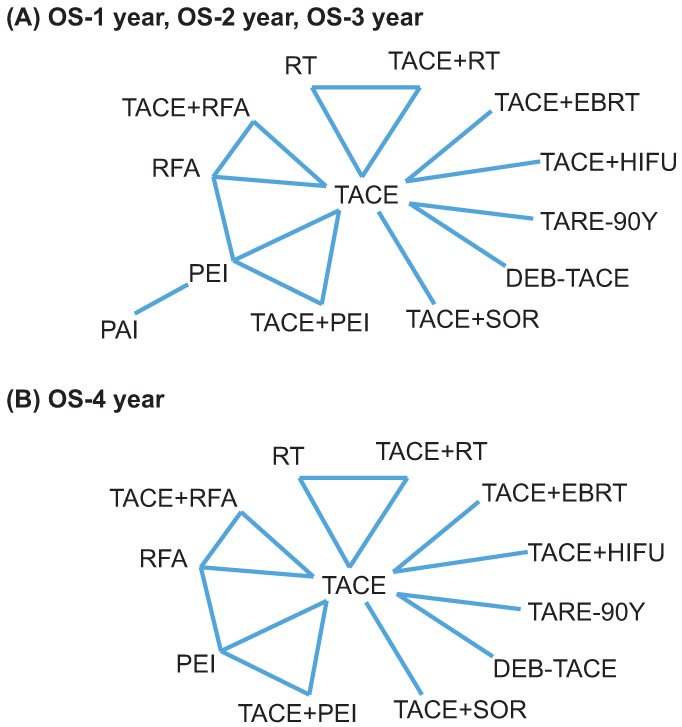
Network design of 13 therapies in the network meta-analysis **A**. OS-1, OS-2, OS-3; **B**. OS-4.

In total 42 articles (Table 1) were selected and included in the study after screening out irrelevant papers [17–58]. Among the total 5,666 subjects, 2,392 (42.22%) individuals underwent TACE treatment and 891 (15.73%) individuals received RFA treatment. Furthermore, 438 (7.73%), 432 (7.62%), 379 (6.69%), 325 (5.74%), 310 (5.47%), 174 (3.07%), 131 (2.31%), 68 (1.20%), 63 (1.11%), 54 (0.95%) and 9 (0.16%) patients underwent TACE+RFA, PEI, TACE + SOR, DEB-TACE, TARE-90Y, TACE + PEI, TACE + RT, TACE + HIFU, PAI, TACE + EBRT and RT respectively. Aside from four trials that were three-arm trials, 38 were two-arm trials and a total of 16 comparisons were created among the 42 studies. In term of OS-1 (Figure 1A), there are 40 studies providing data for 16 comparisons. For OS-2 and OS-3 (Figure 1A), data from 41 and 36 trials are provided, respectively. For OS-4 (Figure 1B), data were provided by 23 studies.

**Table 1:** the main characteristics of included studies

Study	Region	Year	Treatment 1	Treatment2	Size1	Size2	Outcomes
Peng (2013)	China	2013	TACE+RFA	RFA	94	95	1234
Adnan Muhammad (2013)	USA	2013	TACE+SOR	TACE	13	30	1234
Wei Bai (2013)	China	2013	TACE+SOR	TACE	82	164	12
Nicolini (2013)	Italy	2013	DEB-TACE	TACE	22	16	1234
Moreno-Luna (2013)	USA	2013	TARE-90Y	TACE	61	55	1234
Xu-Dong Qu (2012)	China	2012	TACE+SOR	TACE	45	45	1234
Song (2012)	Korea	2012	DEB-TACE	TACE	60	69	123
Recchia (2012)	Italy	2012	DEB-TACE	TACE	35	70	123
Peng (2011)	China	2011	TACE+RFA	RFA	69	70	1234
Masatoshi Kudo (2011)	Japan+South Korea	2011	TACE+SOR	TACE	229	229	123
Song (2011)	Korea	2011	DEB-TACE	TACE	20	20	123
Salem (2011)	USA	2011	TARE-90Y	TACE	123	122	1234
Wiggerman (2011)	Germany	2011	DEB-TACE	TACE	22	22	123
Sacco (2011)	Italy	2011	DEB-TACE	TACE	33	34	123
Malagari (2011)	Greece	2011	DEB-TACE	TACE	41	43	1
Kim (2010)	Korea	2010	TACE+RFA	RFA	83	231	1234
Morimoto (2010)	Japan	2010	TACE+RFA	RFA	19	18	1234
Li (2010)	China	2010	TACE+HIFU	TACE	44	45	1234
Tan (2010)	China	2010	TACE+SOR	TACE	10	10	12
Kooby (2010)	USA	2010	TARE-90Y	TACE	27	44	1234
Carr (2010)	USA	2010	TARE-90Y	TACE	99	691	123
Ferrer Puchol (2010)	Spain	2010	DEB-TACE	TACE	47	25	1234
Dhanasekaran (2010)	USA	2010	DEB-TACE	TACE	45	26	12
Shibata (2009)	Japan	2009	TACE+RFA	TACE	46	43	234
Yang-a (2009)	China	2009	TACE+RFA	RFA	31	37	1234
Yang-c (2009)	China	2009	RFA	TACE	37	35	1234
Yang-b (2009)	China	2009	TACE+RFA	TACE	31	35	1234
Cheng-b (2008)	China	2008	TACE+RFA	TACE	96	95	1234
Cheng-a (2008)	China	2008	TACE+RFA	RFA	96	100	1234
Cheng-c (2008)	China	2008	RFA	TACE	100	95	1234
Brunello (2008)	Italy	2008	RFA	PEI	70	69	1234
Wu (2005)	China	2005	TACE+HIFU	TACE	24	26	12
Becker (2005)	Germany	2005	TACE+PEI	TACE	27	25	123
Shiina (2005)	Japan	2005	RFA	PEI	118	114	1234
Lin (2005)	Taiwan	2005	PAI	PEI	63	62	123
Shim (2005)	Korea	2005	TACE+RT	TACE	38	35	1234
Lin-a (2004)	Taiwan	2004	RFA	PEI	50	46	123
Lin-b (2004)	Taiwan	2004	RFA	PEI	50	50	123
Zeng (2004)	China	2004	TACE+EBRT	TACE	54	149	1234
Lencioni (2003)	Italy	2003	RFA	PEI	52	50	123
Guo (2003)	China	2003	TACE+RT	TACE	76	89	1234
Kamada (2002)	Japan	2002	TACE+PEI	TACE	32	37	1234
Koda (2001)	Japan	2001	TACE+PEI	PEI	26	26	234
Chia-Hsien Cheng-b (2001)	China	2001	TACE+RT	RT	17	9	1234
Chia-Hsien Cheng-c (2001)	China	2001	RT	TACE	9	16	1234
Chia-Hsien Cheng-a (2001)	China	2001	TACE+RT	TACE	17	16	1234
Allgaier-b (1998)	Germany	1998	TACE+PEI	TACE	39	33	12
Allgaier-a (1998)	Germany	1998	TACE+PEI	PEI	39	15	12
Allgaier-c (1998)	Germany	1998	PEI	TACE	15	33	12
Bartolozzi (1995)	Italy	1995	TACE+PEI	TACE	26	27	123
Kato (1994)	Japan	1994	TACE+PEI	TACE	24	22	123

1. TACE: transcatheter arterial chemoembolization; EBRT: external-beam radiation therapy; HIFU: high intensity focused ultrasound; PEI: percutaneous ethanol injection; RFA: radiofrequency ablation; RT: radiotherapy; SOR: sorafenib; TARE-90Y: yttrium-90 radioembolization; DEB-TACE: drug-eluting beads-transcatheter arterial chemoembolization; PAI: percutaneous acetic acid injection.

2. Outcomes: 1-overall survival of 1 year; 2-overall survival of 2 years; 3-overall survival of 3 years; 4-overall survival of 4 years

### Pairwise meta-analysis

We completed the pairwise meta-analysis for the 16 comparisons and the weighted HRs for each comparison was calculated. The results of the pair-wise comparisons is shown in Figure 1 which illustrates the comparison of OS-1, OS-2, OS-3 (Figure 1A) and OS-4 (Figure 1B). For OS-1 (Table 2), direct comparisons suggest that combined TACE therapies, as well as RFA and RT, was more efficacious than TACE monotherapy (all HR < 1 and 95 % CI of HR exclude 1). PEI appeared to be less efficacious than its counterpart TACE + PEI (HR = 2.04, 95% CI, 1.69 - 2.56). Similarly, the efficacy of RFA was worse than that of TACE + RFA (HR = 2.13, 95% CI, 1.92 - 2.44) whereas RFA exhibits better efficacy compared to PEI (HR = 0.30, 95% CI, 0.20 - 0.41).

**Table 2:** Comparing the relative efficacy of therapies with respect to one-year survival status using pairwise and network meta-analysis

	TACE	TACE +EBRT	TACE +HIFU	TACE +PEI	TACE +RFA	TACE +RT	TACE +SOR	TARE -90Y	DEB-TACE	PAI	PEI	RFA	RT
**TACE**		**0.73 (0.56, 0.90)**	**0.24 (0.21, 0.28)**	**0.48 (0.41, 0.54)**	**0.52 (0.39, 0.66)**	**0.52 (0.45, 0.60)**	**0.47 (0.45, 0.49)**	**0.88 (0.80, 0.97)**	**0.51 (0.46, 0.57)**	-	0.91 (0.78, 1.04)	**0.84 (0.67, 1.00)**	**0.42 (0.32, 0.52)**
**TACE+EBRT**	0.73 (0.30, 1.75)		-	-	-	-	-	-	-	-	-	-	-
**TACE+HIFU**	**0.31 (0.17, 0.57)**	0.42 (0.14, 1.22)		-	-	-	-	-	-	-	-	-	-
**TACE+PEI**	0.72 (0.47, 1.09)	0.99 (0.37, 2.60)	**2.35 (1.12, 4.95)**		-	-	-	-	-	-	**2.04 (1.69, 2.56)**		
**TACE+RFA**	**0.45 (0.28, 0.72)**	0.62 (0.23, 1.67)	1.48 (0.68, 3.21)	0.63 (0.34, 1.15)		-	-	-	-	-	-	**2.13 (1.92, 2.44)**	
**TACE+RT**	**0.41 (0.25, 0.66)**	0.56 (0.21, 1.51)	1.33 (0.61, 2.90)	0.56 (0.30, 1.07)	0.90 (0.46, 1.76)		-	-	-	-	-	-	6.25 (5.00, 8.33)
**TACE+SOR**	**0.55 (0.37, 0.83)**	0.76 (0.29, 1.99)	1.80 (0.86, 3.78)	0.77 (0.43, 1.38)	1.22 (0.65, 2.28)	1.36 (0.72, 2.56)		-	-	-	-	-	-
**TARE-90Y**	0.88 (0.57, 1.35)	1.20 (0.45, 3.19)	**2.86 (1.35, 6.07)**	1.22 (0.67, 2.22)	**1.93 (1.02, 3.67)**	**2.15 (1.13, 4.12)**	1.59 (0.87, 2.89)		-	-	-	-	-
**DEB-TACE**	**0.73 (0.53, 1.00)**	0.99 (0.39, 2.53)	**2.37 (1.18, 4.75)**	1.01 (0.59, 1.71)	1.60 (0.91, 2.84)	**1.79 (1.00, 3.19)**	1.32 (0.78, 2.22)	0.83 (0.48, 1.42)		-	-	-	-
**PAI**	1.00 (0.34, 2.91)	1.37 (0.34, 5.44)	3.27 (0.95, 11.19)	1.39 (0.46, 4.19)	2.21 (0.74, 6.63)	2.46 (0.76, 7.93)	1.81 (0.58, 5.69)	1.14 (0.36, 3.61)	1.38 (0.45, 4.20)		1.33 (0.90, 2.56)	-	-
**PEI**	1.33 (0.84, 2.13)	1.83 (0.68, 4.92)	**4.35 (2.01, 9.42)**	**1.85 (1.07, 3.19)**	**2.95 (1.72, 5.04)**	**3.28 (1.68, 6.41)**	**2.42 (1.30, 4.50)**	1.52 (0.80, 2.88)	**1.84 (1.04, 3.24)**	1.33 (0.51, 3.48)		**0.30 (0.20, 0.41)**	-
**RFA**	0.87 (0.57, 1.32)	1.19 (0.45, 3.13)	**2.83 (1.34, 5.95)**	1.20 (0.70, 2.08)	**1.91 (1.27, 2.88)**	**2.13 (1.13, 4.03)**	1.57 (0.87, 2.82)	0.99 (0.54, 1.81)	1.19 (0.70, 2.02)	0.87 (0.31, 2.45)	**0.65 (0.44, 0.97)**		-
**RT**	1.03 (0.53, 2.01)	1.41 (0.47, 4.24)	**3.37 (1.36, 8.34)**	1.43 (0.65, 3.15)	**2.28 (1.01, 5.15)**	**2.54 (1.30, 4.94)**	1.87 (0.85, 4.09)	1.18 (0.53, 2.61)	1.42 (0.68, 2.98)	1.03 (0.29, 3.63)	0.77 (0.34, 1.75)	1.19 (0.54, 2.62)	

1. The cells in blue are results of pair-wise meta-analysis and the column treatment is compared with the row treatment. The results are presented by hazard ratio (HR) and 95% credible interval (CrI). The cells in red are results of network meta-analysis and the row treatment is compared with the column treatment. The results are presented by hazard ratio (HR) and 95% credible interval (CrI).

2. TACE: transcatheter arterial chemoembolization; EBRT: external-beam radiation therapy; HIFU: high intensity focused ultrasound; PEI: percutaneous ethanol injection; RFA: radiofrequency ablation; RT: radiotherapy; SOR: sorafenib; TARE-90Y: yttrium-90 radioembolization; DEB-TACE: drug-eluting beads-transcatheter arterial chemoembolization; PAI: percutaneous acetic acid injection.

For OS-2 (Table 3), the majority of the selected therapies exhibited a greater efficacy compared to TACE monotherapy (HR < 1 and 95 % CI of HR exclude 1). Besides that, PEI monotherapy appeared to be less efficacious than the combined approach of TACE + PEI (HR = 2.02, 95% CI, 1.77 - 2.35). Likewise, patients with RFA exhibited an increased HR compared to those with TACE + RFA (HR = 1.90, 95% CI, 1.66 - 2.22). Finally, RT monotherapy was far less effective than the combined approach of TACE + RT (HR = 5.56, 95% CI, 4.44 - 7.41).

**Table 3:** Comparing the relative efficacy of interventions with respect to two-year survival status using pairwise and network meta-analysis

	TACE	TACE +EBRT	TACE +HIFU	TACE +PEI	TACE +RFA	TACE +RT	TACE +SOR	TARE -90Y	DEB -TACE	PAI	PEI	RFA	RT
**TACE**		**0.71 (0.59, 0.83)**	**0.25 (0.22, 0.28)**	**0.51 (0.46, 0.56)**	**0.53 (0.42, 0.63)**	**0.53 (0.47, 0.59)**	**0.55 (0.50, 0.60)**	**0.87 (0.80, 0.94)**	**0.52 (0.47, 0.57)**	-	-	**0.90 (0.80, 0.99)**	**0.44 (0.34, 0.54)**
**TACE+EBRT**	0.71 (0.33, 1.53)		-	-	-	-	-	-	-	-	-	-	-
**TACE+HIFU**	**0.31 (0.18, 0.54)**	0.44 (0.17, 1.13)		-	-	-	-	-	-	-	-	-	-
**TACE+PEI**	0.76 (0.55, 1.04)	1.07 (0.46, 2.45)	**2.41 (1.28, 4.53)**		-	-	-	-	-	-	**2.02 (1.77, 2.35)**	-	-
**TACE+RFA**	**0.46 (0.31, 0.67)**	0.64 (0.27, 1.52)	1.45 (0.75, 2.83)	**0.60 (0.38, 0.97)**		-	-	-	-	-	-	**1.90 (1.66, 2.22)**	-
**TACE+RT**	**0.44 (0.29, 0.67)**	0.62 (0.26, 1.49)	1.40 (0.71, 2.78)	**0.58 (0.34, 0.99)**	0.96 (0.55, 1.70)		-	-	-	-	-	-	**5.56 (4.44, 7.41)**
**TACE+SOR**	**0.67 (0.47, 0.95)**	0.94 (0.40, 2.18)	**2.12 (1.11, 4.05)**	0.88 (0.55, 1.42)	1.46 (0.87, 2.46)	1.52 (0.88, 2.61)		-	-	-	-	-	-
**TARE-90Y**	0.87 (0.59, 1.28)	1.23 (0.52, 2.90)	**2.78 (1.43, 5.40)**	1.15 (0.70, 1.90)	**1.91 (1.11, 3.29)**	**1.98 (1.12, 3.50)**	1.31 (0.78, 2.20)		-	-	-	-	-
**DEB-TACE**	**0.62 (0.46, 0.84)**	0.87 (0.38, 2.00)	**1.98 (1.06, 3.68)**	0.82 (0.53, 1.27)	1.36 (0.83, 2.22)	1.41 (0.84, 2.36)	0.93 (0.59, 1.47)	0.71 (0.44, 1.16)		-	-	-	-
**PAI**	1.12 (0.46, 2.70)	1.58 (0.49, 5.07)	**3.56 (1.26, 10.03)**	1.48 (0.60, 3.63)	2.45 (0.99, 6.04)	2.54 (0.96, 6.74)	1.68 (0.65, 4.33)	1.28 (0.49, 3.35)	1.80 (0.71, 4.57)		1.78 (0.93, 1.60)	-	-
**PEI**	1.32 (0.90, 1.93)	1.85 (0.79, 4.37)	**4.19 (2.16, 8.13)**	**1.74 (1.15, 2.64)**	**2.88 (1.88, 4.41)**	**2.99 (1.70, 5.26)**	**1.97 (1.18, 3.31)**	1.51 (0.88, 2.59)	**2.12 (1.31, 3.44)**	1.18 (0.53, 2.60)		**0.43 (0.35, 0.50)**	-
**RFA**	0.76 (0.53, 1.08)	1.07 (0.46, 2.49)	**2.42 (1.27, 4.62)**	1.00 (0.65, 1.55)	**1.66 (1.21, 2.28)**	**1.73 (1.00, 2.98)**	1.14 (0.69, 1.87)	0.87 (0.52, 1.47)	1.22 (0.77, 1.94)	0.68 (0.29, 1.61)	**0.58 (0.42, 0.80)**		-
**RT**	1.03 (0.57, 1.86)	1.45 (0.55, 3.82)	**3.27 (1.47, 7.32)**	1.36 (0.69, 2.67)	**2.25 (1.11, 4.57)**	**2.34 (1.29, 4.23)**	1.54 (0.77, 3.07)	1.18 (0.58, 2.39)	1.65 (0.85, 3.21)	0.92 (0.32, 2.66)	0.78 (0.39, 1.58)	1.35 (0.68, 2.70)	

1. The cells in blue are results of pair-wise meta-analysis and the column treatment is compared with the row treatment. The results are presented by hazard ratio (HR) and 95% credible interval (CrI). The cells in red are results of network meta-analysis and the row treatment is compared with the column treatment. The results are presented by hazard ratio (HR) and 95% credible interval (CrI).

2. TACE: transcatheter arterial chemoembolization; EBRT: external-beam radiation therapy; HIFU: high intensity focused ultrasound; PEI: percutaneous ethanol injection; RFA: radiofrequency ablation; RT: radiotherapy; SOR: sorafenib; TARE-90Y: yttrium-90 radioembolization; DEB-TACE: drug-eluting beads-transcatheter arterial chemoembolization; PAI: percutaneous acetic acid injection.

Direct comparisons in Table 4 indicated that TACE + EBRT, TACE + HIFU, TACE + PEI, TACE + RFA, TACE + RT, TACE + SOR, TARE-90Y, DEB-TACE, RFA and RT were more effective than TACE monotherapy with respect to OS-3 (HR < 1 and 95 % CI of HR exclude 1). In addition, patients treated with RFA had higher OS-3compared to those treated with PEI (HR = 0.48, 95% CI, 0.41 - 0.55). On the other hand, RFA was less effective than TACE + RFA (HR = 1.76, 95% CI, 1.56 - 2.00) and RT was less effective than TACE + RT (HR = 5.56, 95% CI, 4.44 - 7.41). The comparison results displayed in Table 5 were very similar to the results mentioned above.

**Table 4:** Comparing the relative efficacy of interventions with respect to three-year survival status using pairwise and network meta-analysis

	TACE	TACE +EBRT	TACE +HIFU	TACE +PEI	TACE +RFA	TACE +RT	TACE +SOR	TARE -90Y	DEB -TACE	PAI	PEI	RFA	RT
**TACE**		**0.69 (0.59, 0.80)**	**0.41 (0.35, 0.48)**	**0.50 (0.43, 0.56)**	**0.56 (0.46, 0.65)**	**0.54 (0.48, 0.60)**	**0.64 (0.56, 0.72)**	**0.84 (0.77, 0.90)**	**0.50 (0.44, 0.56)**	-	-	**0.80 (0.69, 0.91)**	**0.44 (0.34, 0.54)**
**TACE+EBRT**	0.69 (0.32, 1.50)		-	-	-	-	-	-	-	-	-	-	-
**TACE+HIFU**	**0.41 (0.19, 0.90)**	0.59 (0.20, 1.79)		-	-	-	-	-	-	-	-	-	-
**TACE+PEI**	0.77 (0.53, 1.13)	1.12 (0.47, 2.67)	1.89 (0.79, 4.50)		-	-	-	-	-	-	1.39 (0.82, 4.55)	-	-
**TACE+RFA**	**0.52 (0.35, 0.78)**	0.75 (0.31, 1.81)	1.26 (0.52, 3.04)	0.67 (0.39, 1.13)		-	-	-	-	-	-	**1.76 (1.56, 2.00)**	-
**TACE+RT**	**0.45 (0.29, 0.69)**	0.65 (0.27, 1.58)	1.09 (0.45, 2.66)	0.58 (0.33, 1.02)	0.87 (0.48, 1.56)		-	-	-	-	-	-	**5.56 (4.44, 7.41)**
**TACE+SOR**	0.71 (0.45, 1.13)	1.04 (0.42, 2.56)	1.74 (0.71, 4.31)	0.92 (0.51, 1.67)	1.38 (0.75, 2.54)	1.60 (0.85, 2.98)		-	-	-	-	-	-
**TARE-90Y**	0.84 (0.57, 1.25)	1.22 (0.51, 2.93)	2.06 (0.86, 4.93)	1.09 (0.63, 1.88)	1.63 (0.93, 2.86)	**1.89 (1.06, 3.36)**	1.18 (0.65, 2.16)		-	-	-	-	-
**DEB-TACE**	**0.64 (0.46, 0.89)**	0.93 (0.40, 2.16)	1.56 (0.67, 3.64)	0.83 (0.50, 1.36)	1.23 (0.73, 2.08)	1.43 (0.83, 2.44)	0.89 (0.51, 1.57)	0.76 (0.45, 1.26)		-	-	-	-
**PAI**	1.25 (0.49, 3.20)	1.81 (0.53, 6.14)	3.05 (0.90, 10.35)	1.62 (0.61, 4.30)	2.42 (0.96, 6.07)	**2.79 (1.00, 7.83)**	1.75 (0.62, 4.97)	1.48 (0.54, 4.09)	1.96 (0.72, 5.29)		1.14 (0.93, 1.45)	-	-
**PEI**	1.42 (0.86, 2.34)	2.06 (0.82, 5.20)	**3.47 (1.37, 8.76)**	1.84 (1.04, 3.24)	**2.75 (1.73, 4.37)**	**3.17 (1.65, 6.11)**	**1.99 (1.01, 3.91)**	1.68 (0.89, 3.17)	**2.22 (1.22, 4.04)**	1.14 (0.51, 2.52)		**0.48 (0.41, 0.55)**	-
**RFA**	0.81 (0.55, 1.21)	1.18 (0.49, 2.83)	1.99 (0.83, 4.77)	1.05 (0.63, 1.75)	**1.57 (1.15, 2.15)**	**1.82 (1.02, 3.25)**	1.14 (0.62, 2.09)	0.96 (0.55, 1.68)	1.27 (0.76, 2.13)	0.65 (0.27, 1.56)	**0.57 (0.40, 0.82)**		-
**RT**	1.04 (0.57, 1.90)	1.50 (0.56, 4.03)	2.53 (0.94, 6.79)	1.34 (0.66, 2.73)	2.00 (0.97, 4.15)	**2.32 (1.26, 4.24)**	1.45 (0.68, 3.10)	1.23 (0.60, 2.52)	1.62 (0.82, 3.23)	0.83 (0.27, 2.53)	0.73 (0.33, 1.60)	1.27 (0.62, 2.62)	

1. The cells in blue are results of pair-wise meta-analysis and the column treatment is compared with the row treatment. The results are presented by hazard ratio (HR) and 95% credible interval (CrI). The cells in red are results of network meta-analysis and the row treatment is compared with the column treatment. The results are presented by hazard ratio (HR) and 95% credible interval (CrI).

2. TACE: transcatheter arterial chemoembolization; EBRT: external-beam radiation therapy; HIFU: high intensity focused ultrasound; PEI: percutaneous ethanol injection; RFA: radiofrequency ablation; RT: radiotherapy; SOR: sorafenib; TARE-90Y: yttrium-90 radioembolization; DEB-TACE: drug-eluting beads-transcatheter arterial chemoembolization; PAI: percutaneous acetic acid injection.

**Table 5:** Comparing the relative efficacy of therapies with respect to four-year survival status using pairwise and network meta-analysis

	TACE	TACE +EBRT	TACE +HIFU	TACE +PEI	TACE +RFA	TACE +RT	TACE +SOR	TARE -90Y	DEB -TACE	PEI	RFA	RT
**TACE**		**0.69 (0.59, 0.80)**	**0.41 (0.35, 0.48)**	1.29 (0.92, 1.66)	**0.60 (0.50, 0.69)**	**0.55 (0.49, 0.60)**	**0.67 (0.57, 0.76)**	**0.86 (0.79, 0.93)**	0.94 (0.74, 1.14)	-	-	**0.44 (0.34, 0.54)**
**TACE+EBRT**	**0.69 (0.59, 0.80)**		-	-	-	-	-	-	-	-	-	-
**TACE+HIFU**	**0.41 (0.35, 0.48)**	**0.59 (0.48, 0.74)**		-	-	-	-	-	-	-	-	-
**TACE+PEI**	1.19 (0.91, 1.54)	**1.72 (1.27, 2.33)**	**2.90 (2.13, 3.93)**		-	-	-	-	-	1.39 (0.82, 4.54)	-	-
**TACE+RFA**	**0.58 (0.51, 0.65)**	0.84 (0.69, 1.01)	**1.41 (1.16, 1.71)**	**0.49 (0.37, 0.64)**		-	-	-	-	-	**1.59 (1.43, 1.79)**	-
**TACE+RT**	**0.47 (0.43, 0.52)**	**0.68 (0.57, 0.82)**	1.15 (0.95, 1.38)	**0.40 (0.30, 0.52)**	0.81 (0.70, 0.95)		-	-	-	-	-	**5.56 (4.44, 7.41)**
**TACE+SOR**	**0.77 (0.67, 0.88)**	1.11 (0.91, 1.36)	**1.87 (1.52, 2.31)**	**0.65 (0.48, 0.87)**	**1.33 (1.11, 1.59)**	**1.63 (1.38, 1.93)**		-	-	-	-	-
**TARE-90Y**	**0.89 (0.82, 0.96)**	**1.29 (1.08, 1.53)**	**2.17 (1.82, 2.59)**	**0.75 (0.57, 0.98)**	**1.54 (1.33, 1.77)**	**1.89 (1.66, 2.15)**	1.16 (0.99, 1.36)		-	-	-	-
**DEB-TACE**	0.98 (0.79, 1.20)	**1.42 (1.09, 1.83)**	**2.38 (1.83, 3.10)**	0.82 (0.59, 1.15)	**1.69 (1.33, 2.15)**	**2.08 (1.65, 2.62)**	1.27 (0.99, 1.63)	1.10 (0.88, 1.38)		-	-	-
**PEI**	1.08 (0.88, 1.32)	**1.56 (1.21, 2.01)**	**2.63 (2.04, 3.39)**	0.91 (0.67, 1.24)	**1.86 (1.53, 2.27)**	**2.29 (1.83, 2.87)**	**1.40 (1.10, 1.79)**	1.21 (0.98, 1.51)	1.10 (0.83, 1.47)		**0.80 (0.65, 0.94)**	-
**RFA**	**0.85 (0.77, 0.95)**	**1.24 (1.03, 1.49)**	**2.08 (1.72, 2.52)**	**0.72 (0.54, 0.95)**	**1.47 (1.34, 1.62)**	**1.81 (1.57, 2.10)**	1.11 (0.93, 1.32)	0.96 (0.84, 1.10)	0.87 (0.69, 1.10)	**0.79 (0.66, 0.94)**		-
**RT**	0.97 (0.82, 1.15)	**1.40 (1.11, 1.76)**	**2.36 (1.87, 2.98)**	0.81 (0.60, 1.11)	**1.67 (1.36, 2.06)**	**2.06 (1.73, 2.45)**	**1.26 (1.01, 1.57)**	1.09 (0.90, 1.31)	0.99 (0.76, 1.30)	0.90 (0.69, 1.17)	1.13 (0.93, 1.39)	

1. The cells in blue are results of pair-wise meta-analysis and the column treatment is compared with the row treatment. The results are presented by hazard ratio (HR) and 95% credible interval (CrI). The cells in red are results of network meta-analysis and the row treatment is compared with the column treatment. The results are presented by hazard ratio (HR) and 95% credible interval (CrI).

2. TACE: transcatheter arterial chemoembolization; EBRT: external-beam radiation therapy; HIFU: high intensity focused ultrasound; PEI: percutaneous ethanol injection; RFA: radiofrequency ablation; RT: radiotherapy; SOR: sorafenib; TARE-90Y: yttrium-90 radioembolization; DEB-TACE: drug-eluting beads-transcatheter arterial chemoembolization; PAI: percutaneous acetic acid injection.

### Network meta-analysis

As suggested by the lower off-diagonal area in Tables 2, 3, 4, 5, a large number of comparisons were generated by the network meta-analysis. As for OS-1, combined therapies including TACE + HIFU, TACE + RFA, TACE + RT, TACE + SOR and DEB-TACE appeared to be more effective than TACE monotherapy (HR < 1, 95% CrI excludes 1). By contrast, TACE + PEI, TARE - 90Y, DEB - TACE, PEI, RFA and RT were less effective than TACE + HIFU (HR > 1, 95% CrI excludes 1).

Likewise, results from network meta-analysis with respect to OS-2 were displayed in Table 3. Patients treated with combined therapies including TACE + HIFU, TACE + RFA, TACE + RT, TACE + SOR and DEB-TACE were associated with an increased OS-2 in comparison to those treated with TACE monotherapy (HR < 1, 95% CrI excludes 1). Apart from that, TACE + PEI, TACE + SOR, TARE - 90Y, DEB - TACE, PEI, RFA and RT were less effective than TACE+HIFU (HR > 1, 95% CrI excludes 1). Furthermore, patients treated with TACE+RFA and TACE+RT were associated with an increase in OS-2 compared to those treated with TACE+PEI (HR < 1, 95% CrI excludes 1). TARE - 90Y, PEI, RFA and RT appeared to be less effective than TACE + RFA (HR > 1, 95% CrI excludes 1).

For OS - 3 (Table 4), TACE + HIFU, TACE + RFA, TACE + RT and DEB-TACE were associated with an increased OS-3 in comparison to its counterpart TACE (HR < 1, 95% CrI excludes 1). PEI was less effective than TACE + HIFU (HR = 3.47, 95% CrI, 1.37 - 8.76) and both PEI and RFA appeared to be less effective than TACE + RFA (HR = 2.75 95%, CrI: 1.73 - 4.37; HR = 1.57 95%, CrI: 1.15 - 2.15). TARE - 90Y, PAI, PEI, RFA and RT were less effective than TACE + RT (HR > 1, 95% CrI excludes 1).

Comparisons among therapies with respect to OS-4 were displayed in Table 5. Therapies including TACE + EBRT, TACE + HIFU, TACE + RFA, TACE + RT, TACE + SOR, TARE - 90Y and RFA were superior to TACE monotherapy (all HR < 1, 95% CrI excludes 1). TACE + PEI, TACE + RT, TARE - 90Y, DEB - TACE, PEI, RFA and RT were less effective than TACE + EBRT (HR > 1, 95% CrI excludes 1). A few therapies appeared to be less effective than TACE + HIFU including TACE + PEI, TACE + RFA, TACE + SOR, TARE - 90Y, DEB - TACE, PEI, RFA and RT (HR > 1, 95% CrI excludes 1). TACE + RFA, TACE + RT, TACE + SOR, TARE - 90Y and RFA exhibited enhanced efficacy compared to TACE + PEI (HR < 1, 95% CrI excludes 1). TACE + SOR, TARE - 90Y, DEB - TACE, PEI, RFA and RT were less effective than TACE + RFA (HR > 1, 95% CrI excludes 1).

**Figure 2:**
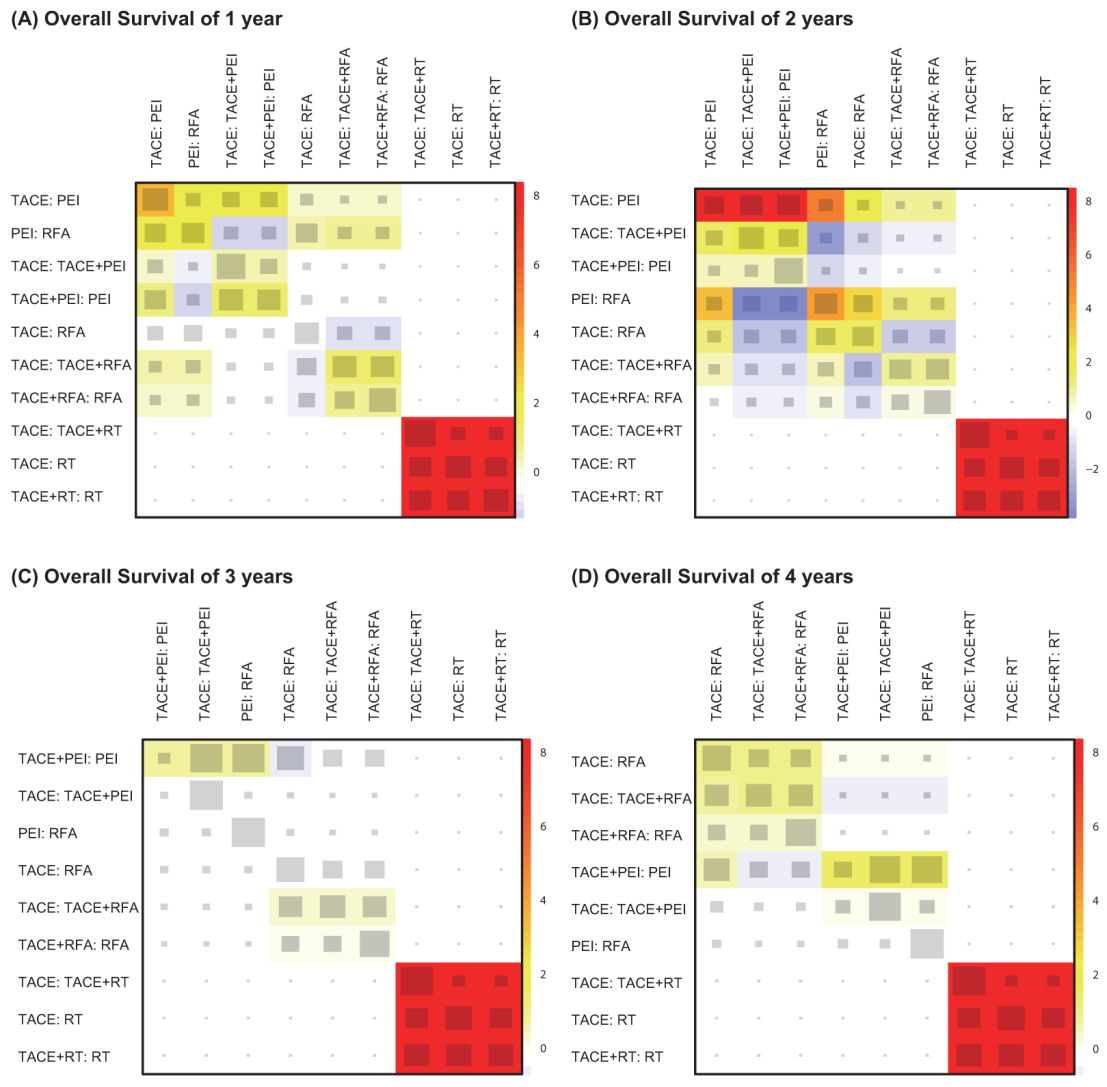
Net heat plots for different study designs revealing changes in inconsistency between direct and indirect evidence **A**. OS-1; **B**. OS-2; **C**. OS-3; **D**. OS-4.

Since the reliability of network meta-analysis can be assessed by consistency between direct and indirect evidence, we also obtained a net heat plot to achieve that purpose (Figure 2). The horizontal and vertical axis corresponds to evidence of study designs obtained from direct and indirect comparisons, respectively. The size of the square indicates the contribution of direct estimates in a specific design in relation to the network estimate in the corresponding design. In other words, the size of square suggests the extent of direct estimates of study designs contributed to the corresponding mixed estimates. In Figure 2A, designs including TACE: PEI, TACE: TACE + PEI, TACE + RFA: RFA and TACE: TACE + RT had substantial effects on the corresponding network estimates. The corresponding colors in the net heat plot reveal changes in inconsistency between direct and indirect evidence in a study design once direct evidence of this design is detached. Blue colors suggest an increase in inconsistency whereas warm colors suggest a decrease in inconsistency. The strongest reduction in inconsistency resulted from the detachment of studies TACE: TACE + RT, TACE: RT, TACE: PEI and TACE + RT: RT since they appeared to have the most intensive colors (Figure 2A). This pattern was also replicated in Figure 2B to 2D. Therefore, study designs corresponding to TACE: TACE + RT, TACE: RT and TACE + RT: RT appeared to have strong inconsistency between direct and indirect evidence since there was significant reduction in inconsistence once we detached the corresponding direct evidence of these studies.

**Figure 3:**
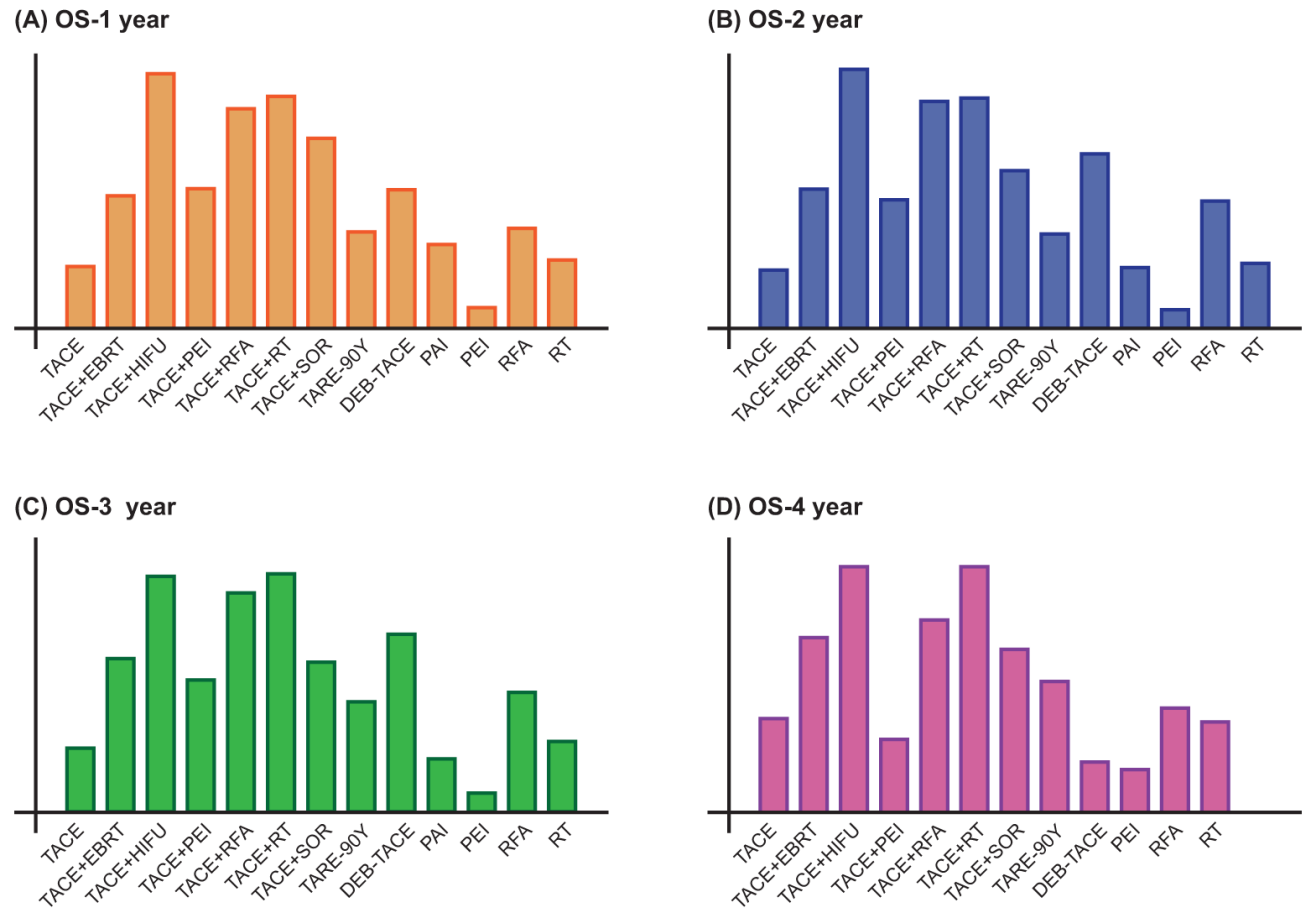
Surface under the cumulative ranking curve (SUCRA) of different interventions with respect to **A**. OS-1; **B**. OS-2; **C**. OS-3; and **D**. OS-4.

Finally, Figure 3 provides the ranking probabilities of these interventions based on their corresponding SUCRA values. TACE + HIFU exhibited the most promising result whereas PEI has the least efficacy with respect to OS-1 year (Figure 3A). Similar rankings were displayed in Figure 3B-3D, revealing that TACE + HIFU had largest efficacy whereas PEI was the least efficacious one. Moreover, combined treatments including TACE + RFA and TACE + RT provided patients with almost equivalent efficacy in comparison to TACE + HIFU. Aside from that, introducing other therapies (EBRT, HIFU, RFA, RT, and SOR) into TACE substantially enhanced the efficacy of TACE

## DISCUSSION

TACE has been widely introduced to HCC patients when surgical resection is believed to be inappropriate [59]. Some detractors, however, have voiced that TACE should not be carried out for patients with portal vein tumor thrombus (PVTT) as it may result in ischemic liver damage under certain circumstances [60]. As suggested by both the pair-wise meta-analysis and network meta-analysis in our study, TACE + HIFU appeared to be far more effective than TACE monotherapy over the four-year period. HIFU is usually combined with TACE in order to induce complete tumor necrosis which is not achievable by using TACE monotherapy [14]. A prospective randomized trial indicated that patients who underwent TACE + HIFU exhibited significantly better responses than those who underwent repeated segmental TACE monotherapy [56]. HIFU ablation is an effective therapy which is characterized by non-invasiveness and it is particularly suited for treating localized tumors [61]. The combined therapy of HIFU + TACE reduced the side effects resulted from the use of repeated TACE without significant efficacy loss.

In our study, the combined therapy of TACE and RFA also exhibited promising results and an average of 50% reduction in the HR was suggested in comparison to those with TACE. Two common combined approaches have been introduced into clinical practices. The first approach is commonly carried out by firstly implementing TACE followed by RFA. The second approach is conducted in the reverse order. Choosing an appropriate time interval between TACE and RFA also has a significant impact on the efficacy and safety [62]. For instance, liver functions are more likely to be preserved if sufficient time is allowed between the implementation of TACE and RFA [63]. However, an extended time between TACE and RFA may increase the number of treatment days along with the associated administration costs. On the other hand, a relatively short interval can contribute to stronger efficacy as the synergistic effects induced by TACE and RFA are likely to be increased. Nevertheless, such an increase in the synergistic effects may also be associated with a potential increase in the risk of liver dysfunction which is commonly observed in cirrhotic patients [62]. As a result, the sequence in which TACE and RFA are carried out as well as the optimal interval between these therapies must be determined before the implementation. Recently, some encouraging results have been obtained, indicating that TACE followed by RT is able to improve the survival status and the local response rate of unresectable HCC patients [64, 65]. Several advantages of combining TACE with RT have been mentioned in the current literature. For instance, TACE may trigger the decrease of tumor volume and such a decrease may contribute to the increase in the radiation dose delivered to the target tumor [66].

On the other hand, our study indicated that PEI was considered to be the one with the least compelling results. The implementation of PEI is quite simple and it involves injecting ethanol into the tumor mass for inducing coagulative necrosis of neoplastic nodules. Unlike TACE, the side effects resulted from PEI is relatively small even if procedures are repeated several times. The less compelling results of PEI may be explained by the fact that the volume of ethanol injection plays a critical role in its efficacy and the corresponding volume of injection is determined by the maximum radius of the lesion [67]. In view of the advantages of the PEI and to enhance its efficacy, PEI combined with other interventions has been studied. For instance, incorporating TACE into PEI yielded a higher complete response rate and more favorable survival status [31, 68].

There are several limitations to be noted in our study. Firstly, we extracted survival data from individual studies and some data was estimated using the survival function if the original data was not available. Estimating survival data can be challenging, particularly the censoring status since it can only be obtained from the original data and the lack of censoring status will have a significant impact on the characteristics of the corresponding survival functions. Secondly, the sequence in which combined therapies were conducted may influence the results and our study did not allow us to assess the sequences due to the nature of the meta-analysis. The implementation interval between the two procedures and the volume of ethanol injection were rarely revealed from studies which prevented us to evaluate how these two potential factors were linked with efficacy and safety of the corresponding therapies.

Overall, this network meta-analysis concluded that pairwise combination of TACE + HIFU, TACE + RFA and TACE + RT exhibited strong efficacy for unresectable HCC patients whereas PEI seems to be less effective than other standard or combined therapies.

## MATERIALS AND METHODS

### Search strategy

We searched for randomized control trials with the following search terms: “transcatheter arterial chemoembolization” (TACE) or “drug-eluting beads-trans -catheter arterial chemoembolization” (DEB-TACE) or “yttrium-90 radioembolization” (TARE-90Y) or “percutaneous ethanol injection” (PEI) or “radiofrequency ablation” (RFA) or “radiotherapy” (RT) or “percutaneous acetic acid injection” (PAI) or “high intensity focused ultrasound” (HIFU) or “external-beam radiation therapy” (EBRT ) or “sorafenib” (SOR) matched with “hepatocellular carcinoma” in PubMed, Embase, and China National Knowledge Internet (CNKI), which were updated on April 1, 2016, without restrictions on language. A manual search was also performed on the reference list of each relevant study. Previous meta-analyses and earlier reviews of intravesical instillation therapies in HCC were also reviewed to avoid any omissions. Individually, two reviewers performed the research and literature retrieval. All the arguments were solved with a mediating third reviewer.

### Inclusion and exclusion criteria

Studies were eligible if: (1) were categorized as RCTs; (2) patients involved had HCC with a pathologic diagnosis and were over 18 years of age; (3) studies focused on the comparative efficacy of at least two standard or combined minimally invasive procedures (TACE, TACE + SOR, TACE + HIFU, TACE + PEI, DEB-TACE, TARE-90Y, TACE + EBRT, TACE + RT, TACE + RFA, RFA, PEI, PAI); (4) studies compared the efficacy between radiotherapy and minimally invasive procedures; (5) no surgical intervention had been performed on patients; (6) sufficient data were provided within the study. Articles were excluded if: (1) patients involved had multiple concurrent diseases, for instance, coronary artery disease as concomitance; (2) patients had received surgery, drugs, radiotherapy or other treatment approaches before study commencement; (3) there were no sufficient data provided by the study.

### Outcome measures and data extraction

The following data were extracted from eligible studies: gender, patient age, sample size, duration of follow-up, and type of treatment. Two investigators reviewed the manuscripts of all the eligible studies and extracted data into a database independently. Other retrieved data included study duration, disease site, treatment protocols, study location and the number of patients in each arm. The efficacy of the corresponding therapies was measured by using multiple survival rates (overall survival of 1 year, OS-1; overall survival of 2 years, OS-2; overall survival of 3 years, OS-3; overall survival of 4 years, OS-4). Data extraction was conducted by two reviewers independently.

### Statistical analysis

We initially carried out a conventional pair-wise meta-analysis which directly compares each pair of treatments. The corresponding hazard ratios (HRs) and 95% confidence intervals (CI) for each study were pooled in order to obtain the overall effect size. Furthermore, a network meta-analysis was performed for each endpoint with a Bayesian framework using R 3.2.3 software. Both direct and indirect evidence was synthesized in order to compare the efficacy which was assessed using the HRs and 95% credible intervals (CrI). Net heat plots were used to visually assess the degree of inconsistency between direct and indirect evidence. Then the cumulative ranking curve (SUCRA) was created to rank the standard or combined minimally invasive procedures with respect to their efficacy. The ranking probabilities were defined as cumulative probabilities that each therapy being ranked as the first, second and so on. For each endpoint, a therapy is more desirable than others if it has a larger SUCRA value.
